# Supplementary material for: SQANTI-SIM: a simulator of controlled transcript novelty for lrRNA-seq benchmark
Source: Genome Biol. 2023 Dec 11;24:286. doi: 10.1186/s13059-023-03127-0 (PMC10712166; doi:10.1186/s13059-023-03127-0)
Supplement: Supplementary file 1 — Additional file 1: Table S1. Requested and simulated transcript models and read counts for ONT and PacBio datasets used in SQANTI-SIM validation. Table S2 Simulated cDNA ONT and PacBio datasets used for pipeline benchmarking. [file 13059_2023_3127_MOESM1_ESM.pdf]

ADDITIONAL FILE 1: Supplementary tables

SQANTI-SIM: a simulator of controlled transcript novelty for lrRNA-seq benchmark

Jorge Mestre-Tomás, Tianyuan Liu, Francisco J. Pardo-Palacios, Ana Conesa

Table S1 Requested and simulated transcript models and read counts for ONT and PacBio datasets used in SQANTI-SIM validation.

Structural	Requested			Simulated		
category	Isoforms	ONT reads	PacBio reads	Isoforms	ONT reads	PacBio reads
FSM	43000	5377592	745678	43000	5377592	745678
ISM	1000	31129	11894	1000	31129	11894
NIC	1000	29911	10443	1000	29911	10443
NNC	1000	24630	11730	1000	24630	11730
Genic Genomic	1000	27806	12563	1000	27806	12563
Antisense	1000	29078	11982	1000	29078	11982
Fusion	1000	27960	12963	1000	27960	12963
Intergenic	1000	28270	12057	1000	28270	12057

Table S2 Simulated cDNA ONT and PacBio datasets used for pipeline benchmarking.

Simulation	ONT dataset	PacBio dataset
Known transcripts	35,128	35,028
ISM transcripts	5,000	5,000
NIC transcripts	5,000	5,000
NNC transcripts	5,000	5,000
Known read count	18,507,455	3,038,634
Novel read count	1,501,250	854,255
No. of short reads	39,994,086	39,996,784
CAGE peaks	39,198	52,295
