# Supplementary material for: SQANTI-SIM: a simulator of controlled transcript novelty for lrRNA-seq benchmark
Source: Genome Biol. 2023 Dec 11;24:286. doi: 10.1186/s13059-023-03127-0 (PMC10712166; doi:10.1186/s13059-023-03127-0)
Supplement: Supplementary file 2 — Additional file 2:. Figure S1. Number of detected true (TP) and false positives (FP) for different types of novelty (ISM, NIC, and NNC). Figure S2. Relationship between true positives (TP), false negatives (FN), and false positives (FP) with (a) transcript length, (b) number of exons, and (c) simulated expression level. Figure S3. SQANTI-SIM characterization of CAGE peak data. [file 13059_2023_3127_MOESM2_ESM.pdf]

ADDITIONAL FILE 2: Supplementary figures

SQANTI-SIM: a simulator of controlled transcript novelty for lrRNA-seq benchmark

Jorge Mestre-Tomás, Tianyuan Liu, Francisco J. Pardo-Palacios, Ana Conesa

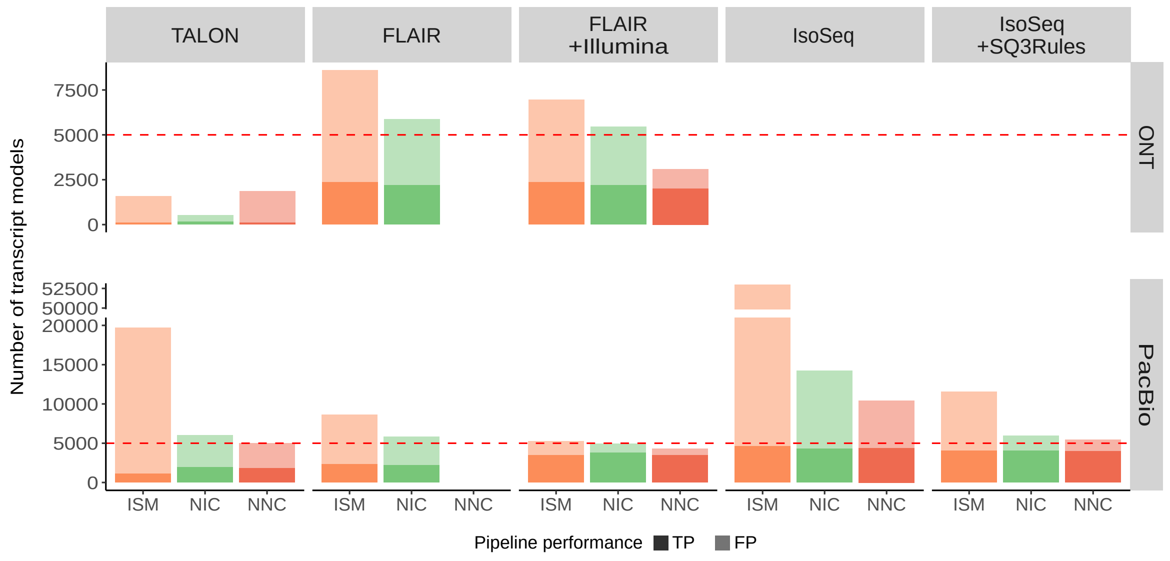

Fig. S1 Number of detected true (TP) and false positives (FP) for different types of novelty (ISM, NIC, and NNC).

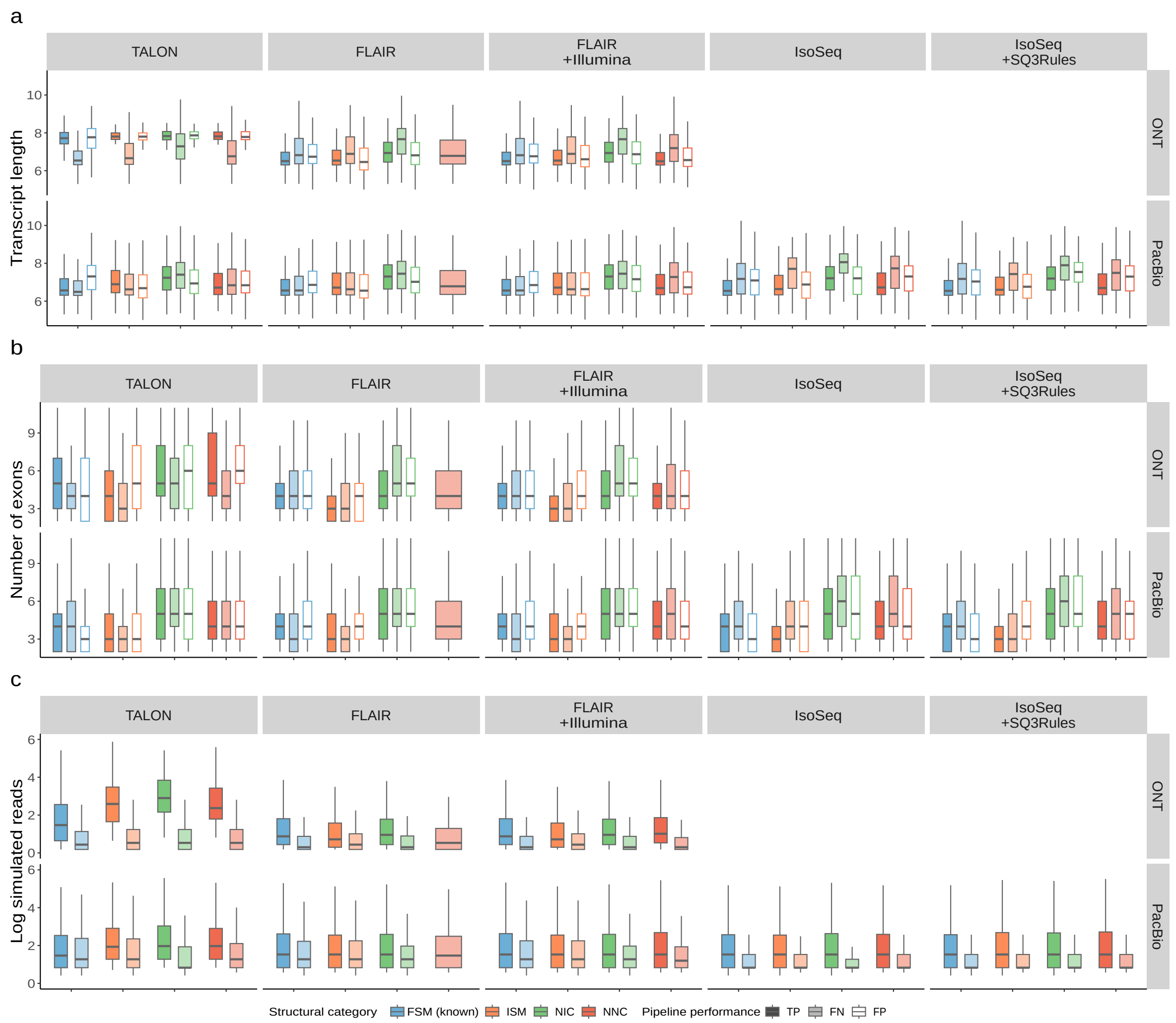

Fig. S2 Relationship between true positives (TP), false negatives (FN), and false positives (FP) with (**a**) transcript length, (**b**) number of exons, and (**c**) simulated expression level.

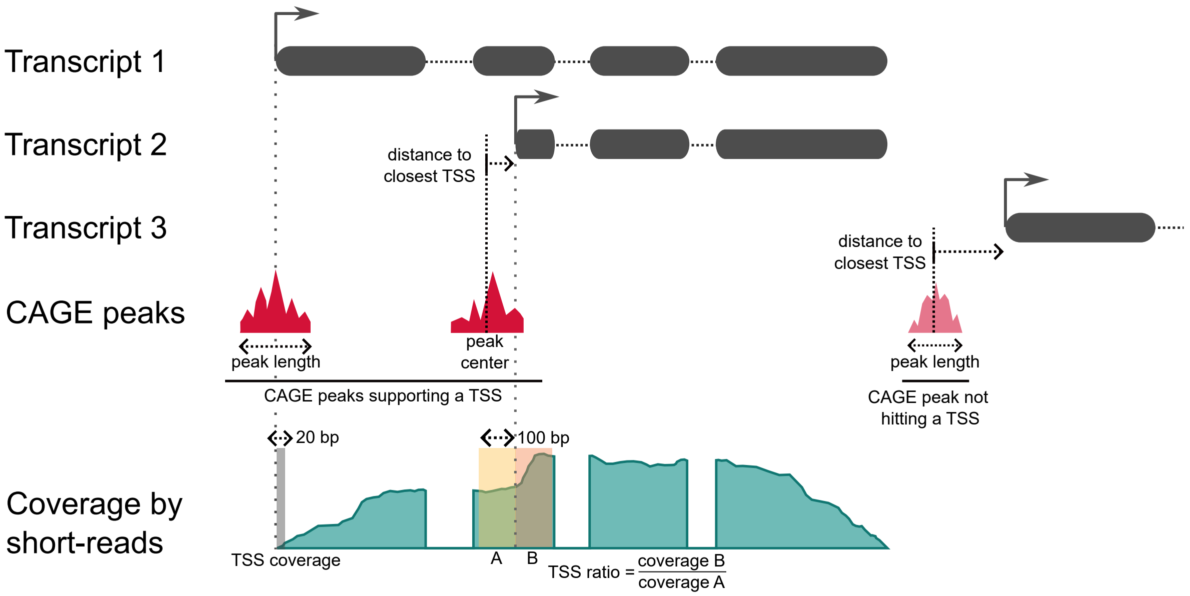

Fig. S3 SQANTI-SIM characterization of CAGE peak data.
