# Supplementary material for: SQANTI-SIM: a simulator of controlled transcript novelty for lrRNA-seq benchmark
Source: Genome Biol. 2023 Dec 11;24:286. doi: 10.1186/s13059-023-03127-0 (PMC10712166; doi:10.1186/s13059-023-03127-0)
Supplement: Supplementary file 4 — Additional file 4. Peer review history. [file 13059_2023_3127_MOESM4_ESM.docx]

**Review history**

**First round of review**

**Reviewer 1**

In the manuscript "SQANTI-SIM: a simulator of controlled transcript novelty for lrRNA-seq benchmark", Mestre-Tomás et al. introduce the SQUANTI-SIM tool. SQUANTI-SIM aims to enable more comprehensive benchmarking of long-read isoform reconstruction tools like FLAIR or Bambu. In particular it aims to address the complication of simulating reads based on isoforms in a reference annotation while also generating new, unannotated isoforms. SQUANTI-SIM takes a smart approach by evaluating isoform structural categories in the reference annotation and then removing specific isoforms from that reference annotation. The reduced reference annotation is then used as input in off the shelf long-read simulation tools. SQUANTI-SIM also simulates short reads and CAGE peaks which can be used as input into isoform reconstruction tools or for further validation.

Overall, I think this is a great tool addressing a real need. Benchmarking tools on novel transcripts is challenging and SQUANTI-SIM adds a missing piece to that puzzle. I don't have any major concerns with the publication of this manuscript, just a few suggestions.

1) Looking at the SQANTI-SIM github, the need to run several subcommands doesn't necessarily scream ease-of-use. But since these type of read simulations come with a lot of parameters, that might be unavoidable. What I would suggest however is for the authors to include a few mouse and human subsampled gtfs with different levels of novelty and associated simulated reads. Considering the extensive expertise of the authors in tool benchmarking I'd leave it to them to choose parameters. I understand that these types of datasets can be large and might be a challenge to host but I think they would be very useful for tool developers to get an easy start with the tool and a preview of its output.

2) I would love some clarification on how isoforms are chosen to be removed from a reference and how the removal doesn't change the categories of other isoforms that remain. For example, the removal of a FSM isoform might turn an ISM isoform into a FSM isoform if the ISM isoform was originally contained within the FSM isoform. I hope this makes sense. In general, how are the relationships between isoforms handled. I think this is briefly addressed in line 740 with "while retaining the associated reference gene/transcript information to preserve the previously assigned structural category" but I'd just like for this to be expanded upon.


Minor points:

Line 305: 4300 should probably read 43000
In 4c, it looks like the axes might be switched. There shouldn't be more mapped than simulated reads. I might be misreading it though.
In 2e the colors of the stacked bars are confusing. There are colors in the legend that are not in the bars and colors in the bars that are not in the legend.

**Reviewer 2**

The authors present a tool to simulate novel transcripts to provide benchmarks with truth sets for long-read transcript annotation tools. This is done by identifying existing transcripts with desired features and removing them from the annotation GFF. The reads are then simulated from these removed transcripts and various tools can be evaluated based on their ability to discover the removed, now novel, transcripts. The authors benchmark several tools for their ability to discover novel transcripts. I found the manuscript to be well written and easy to follow. I was also able to install and run the software on the provided example dataset and there is a large amount of documentation to help users get started. I only have some minor comments:

- Some dependencies are not listed on the wiki page (but are in the conda dependencies) such as bcbio-gff, pybedtools, and gffread
- The simulated transcripts are limited to those known in the existing annotation. Have the authors considered using some updated gene sets (for example from CHM13 T2T) as those may reflect features/transcripts not present in existing annotation sets/genomes
- Would it be possible to use existing rather than simulated reads as well? Since the GFF is reduced but the reads from those transcripts should still be present in a real dataset, the tools should be able to reconstruct them. The same tools used to evaluate their coverage/etc would still work?
- The mappings parameters for minimap2 mention map-pb but, since these reads HiFi, wouldn't map-hifi be more appropriate?

**Authors’ response to reviewers**

**Comments from Reviewer 1**

In the manuscript "SQANTI-SIM: a simulator of controlled transcript novelty for lrRNA-seq benchmark", Mestre-Tomás et al. introduce the SQUANTI-SIM tool. SQUANTI-SIM aims to enable more comprehensive benchmarking of long-read isoform reconstruction tools like FLAIR or Bambu. In particular it aims to address the complication of simulating reads based on isoforms in a reference annotation while also generating new, unannotated isoforms. SQUANTI-SIM takes a smart approach by evaluating isoform structural categories in the reference annotation and then removing specific isoforms from that reference annotation. The reduced reference annotation is then used as input in off the shelf long-read simulation tools. SQUANTI-SIM also simulates short reads and CAGE peaks which can be used as input into isoform reconstruction tools or for further validation.

Overall, I think this is a great tool addressing a real need. Benchmarking tools on novel transcripts is challenging and SQUANTI-SIM adds a missing piece to that puzzle. I don't have any major concerns with the publication of this manuscript, just a few suggestions.

# Many thanks for your comments.

1) *Looking at the SQANTI-SIM github, the need to run several subcommands doesn't necessarily scream ease-of-use. But since these type of read simulations come with a lot of parameters, that might be unavoidable. What I would suggest however is for the authors to include a few mouse and human subsampled gtfs with different levels of novelty and associated simulated reads. Considering the extensive expertise of the authors in tool benchmarking I'd leave it to them to choose parameters. I understand that these types of datasets can be large and might be a challenge to host but I think they would be very useful for tool developers to get an easy start with the tool and a preview of its output.*

**Thank you for your suggestion; we appreciate your feedback. We understand the concern about multiple subcommands; simulation tools often involve many parameters. We're committed to enhancing user support with more example datasets and comprehensive guidance for beginners. We already have a starting point with a toy dataset and two working examples for human on our Wiki (**[**https://github.com/ConesaLab/SQANTI-**](https://github.com/ConesaLab/SQANTI-SIM/wiki/Tutorial) [**SIM/wiki/Tutorial**](https://github.com/ConesaLab/SQANTI-SIM/wiki/Tutorial)**). As suggested, we've also added a subsampled GTF for mouse data on GitHub, along with a tutorial on our Wiki.**

2) *I would love some clarification on how isoforms are chosen to be removed from a reference and how the removal doesn't change the categories of other isoforms that remain. For example, the removal of a FSM isoform might turn an ISM isoform into a FSM isoform if the ISM isoform was originally contained within the FSM isoform. I hope this makes sense. In general, how are the relationships between isoforms handled. I think this is briefly addressed in line 740 with "while retaining the associated reference gene/transcript information to preserve the previously assigned structural category" but I'd just like for this to be expanded upon.*

**Certainly, we appreciate your request for clarification. We have added the following text: “Transcripts chosen for removal, simulated as novel, maintain their assigned structural category by preserving the associated reference transcript within the reduced annotation. Meanwhile, the transcripts not deleted will be simulated as known and thus classified as FSM.”. To ensure the proper functioning of this process, we've established a validation check as illustrated in Figure 2a.**

**For a practical example, if we initially classify Transcript2 as an ISM originating from Transcript1 in the first stage (sqanti-sim.py classif):**

**Transcript 1. 5' [====]----[====]----[====]----[====] 3'**

**Transcript 2. 5' [====]----[====]----[====]	3'**

**If we then remove Transcript2 to simulate an ISM novel transcript, it is crucial to retain Transcript1 in the reduced reference. This ensures that the reconstructed novel transcript (model 2) will continue to be classified as an ISM.**

Minor points:

*Line 305: 4300 should probably read 43000* **We have modified the text.**

In 4c, it looks like the axes might be switched. There shouldn't be more mapped than simulated reads. I might be misreading it though.

# We appreciate your keen observation. You are absolutely correct; in principle shouldn't be more mapped than simulated reads, although for specific genes this might happen because of missmapping associated with mapping errors. We confirm that the axes are indeed correct. While there are a few points above the diagonal line for low counts in 2c, the majority of points are denser below the line, indicating that fewer reads are mapped than simulated. To improve interpretation, we have added the number of reads above and under the diagonal. You can appreciate that most of them are indeed under. Thank you for bringing this to our attention.

In 2e the colors of the stacked bars are confusing. There are colors in the legend that are not in the bars and colors in the bars that are not in the legend.

# Thanks for bringing this legend to our attention. We have corrected the colors.

**Comments from Reviewer 2**

The authors present a tool to simulate novel transcripts to provide benchmarks with truth sets for long- read transcript annotation tools. This is done by identifying existing transcripts with desired features and removing them from the annotation GFF. The reads are then simulated from these removed transcripts and various tools can be evaluated based on their ability to discover the removed, now novel, transcripts. The authors benchmark several tools for their ability to discover novel transcripts. I found the manuscript to be well written and easy to follow. I was also able to install and run the software on the provided example dataset and there is a large amount of documentation to help users get started. I only have some minor comments:

- *Some dependencies are not listed on the wiki page (but are in the conda dependencies) such as bcbio- gff, pybedtools, and gffread*

# Thank you not only for reviewing the manuscript but also for taking the time to examine the documentation and test the tool. We've addressed the missing dependencies you mentioned.

- *The simulated transcripts are limited to those known in the existing annotation. Have the authors considered using some updated gene sets (for example from CHM13 T2T) as those may reflect features/transcripts not present in existing annotation sets/genomes*

# SQANTI-SIM is designed to work with any provided reference genome and annotation and simulates known and novel transcripts by working with the provided gtf. Therefore, it should also work with CHM13 T2T and use these annotations for simulating the novelty. The simulated transcripts would be selected based on the new reference used for simulation.

- *Would it be possible to use existing rather than simulated reads as well? Since the GFF is reduced but the reads from those transcripts should still be present in a real dataset, the tools should be able to reconstruct them. The same tools used to evaluate their coverage/etc would still work?*

# To ensure a comprehensive evaluation, it is essential to have a ground truth, and that is exactly why we simulate data. Although we use real reads to calculate the expression distribution, and the wrapped simulators make use of pre-trained read models, the difficulty lies in obtaining this critical ground truth from real datasets, as in this case we do not know which transcripts are expressed. Therefore, data simulation is crucial to establish the accuracy of any benchmark.

- *The mappings parameters for minimap2 mention map-pb but, since these reads HiFi, wouldn't map- hifi be more appropriate?*

**The mapping step in SQANTI-SIM occurs during the design stage to obtain the expression distribution of a real sample in FASTA or FASTQ format. Currently, SQANTI-SIM uses the "map-pb" parameter by default. However, in response to the increasing use of HiFi reads, we welcome your suggestion to change to "map-hifi". To increase flexibility, we now allow users to enter pre-aligned files in SAM format, which will allow them to map the reads using their preferred approach. Thank you for your valuable input.**
